# Developing expertise in bioinformatics for biomedical research in Africa

**DOI:** 10.1016/j.atg.2015.10.002

**Published:** 2015-10-22

**Authors:** Thomas K. Karikari, Emmanuel Quansah, Wael M.Y. Mohamed

**Affiliations:** a Neuroscience, School of Life Sciences, University of Warwick, Coventry CV4 7AL, UK; b Midlands Integrative Biosciences Training Partnership, University of Warwick, Coventry CV4 7AL, UK; c Pharmacology, Faculty of Health and Life Sciences, De Montfort University, Leicester LE1 9BH, UK; d Department of Molecular Biology and Biotechnology, School of Biological Science, University of Cape Coast, Cape Coast, Ghana; e Clinical Pharmacology Department, Menoufia Medical School, Menoufia University, Egypt

**Keywords:** Africa, Bioinformatics, Computational biology, Biomedical research, Genomics, Research capacity, ASBCB, African Society for Bioinformatics and Computational Biology, FOSRs, free and open source resources, H3ABioNet, a pan-African bioinformatics network funded under the H3Africa project, H3Africa, Human Heredity of Health in Africa, ISCB, International Society for Computational Biology, EVD, Ebola virus disease

## Abstract

Research in bioinformatics has a central role in helping to advance biomedical research. However, its introduction to Africa has been met with some challenges (such as inadequate infrastructure, training opportunities, research funding, human resources, biorepositories and databases) that have contributed to the slow pace of development in this field across the continent. Fortunately, recent improvements in areas such as research funding, infrastructural support and capacity building are helping to develop bioinformatics into an important discipline in Africa. These contributions are leading to the establishment of world-class research facilities, biorepositories, training programmes, scientific networks and funding schemes to improve studies into disease and health in Africa. With increased contribution from all stakeholders, these developments could be further enhanced. Here, we discuss how the recent developments are contributing to the advancement of bioinformatics in Africa.

## 1 Introduction

Advances in bioinformatics are helping the biomedical research community to obtain deeper insights into the fundamentals of biology, through advanced technologies such as high-throughput genomic sequencing and its data analysis, as well as mathematical modelling of biological processes (Karikari, 2015a, Karikari and Aleksic, 2015). While this area of research has been prominent in many scientifically-advanced continents, it was, until recently, not well developed in many emerging regions including Africa (Bishop et al., 2015, Masiga and Isokpehi, 2004). Although bioinformatics holds the potential of helping to bring state-of-the-art biological research to many parts of Africa, its introduction to the continent has been met with challenges such as the low availability of: training programmes, research facilities, expert scientists and research funding (Bishop et al., 2015, Karikari, 2015a, Karikari and Aleksic, 2015, Ojo and Omabe, 2011). These shortcomings have had negative consequences on genomic, genetic and other health-related research across the continent (Bishop et al., 2015, H3Africa Consortium et al., 2014). Consequently, many African scientists have been left unequipped to participate fully in bioinformatics research (Bishop et al., 2015, Karikari, 2015a, Ojo and Omabe, 2011). While Africa remains one of the most genetically-diverse continent on the globe, comprehensive high-quality genomics and genetics studies conducted exclusively on African populations have been lacking (Gomez et al., 2014, H3Africa Consortium et al., 2014). Moreover, there is a low application of genomic technologies for clinical use (Karikari and Aleksic, 2015, Quansah and Karikari, 2015).

Recent improvements in bioinformatics capacity-building through activities such as research funding and training opportunities have made important contributions to developing bioinformatics research in Africa (Adoga et al., 2014, H3Africa Consortium et al., 2014, Karikari, 2015a). These support programmes are boosting scientific capacity for world-class bioinformatics research in Africa. Here, we outline these developments and discuss how they are helping to bring a transformation in bioinformatics to Africa.

## 2 Research infrastructure

Although bioinformatics usually requires considerably less infrastructural investments compared to bench science-intensive disciplines, essential resources such as powerful computer systems, reliable high-speed Internet, access to essential databases and software programmes, and reliable electricity supply are necessary (Karikari, 2015a, Ojo and Omabe, 2011). Presently, the challenge regarding access to computers and the Internet in Africa is gradually fading away, thus allowing more African researchers to improve their usage of Internet-based resources to advance research activities (Ojo and Omabe, 2011). The application of cloud-based web services is also gaining popularity for virtual storage of, and remote access to, data (Bishop et al., 2015). Additionally, the usefulness of low-cost open source technologies and mini-computer systems is becoming evident for teaching and research (Baden et al., 2015, Karikari, 2015a). These developments have positive implications for collaborative research and student training. Furthermore, cost reductions in high-throughput sequencing and other advanced technologies would allow more African laboratories to acquire and apply these resources to improve their research (Karikari, 2015a).

Moreover, the establishment of new research centres incorporating bioinformatics resources as well as new bioinformatics departments in existing institutes is helping to increase participation in bioinformatics and providing opportunities for more people to be trained in this discipline (Table 1). These research facilities do play crucial roles in training the next generation of researchers while also providing employment opportunities for young scientists (Karikari, 2015a). These investments in physical and intellectual resources are making significant contributions to advancing biomedical research through bioinformatics application.

An important resource in biomedical research is biorepositories where samples can be stored and retrieved when needed. However, such repositories are lacking in many parts of Africa, and this negatively affects sample sharing (Adoga et al., 2014). In order to improve this situation, H3Africa is funding the establishment of biorepositories that will store samples obtained in the H3Africa project (Adoga et al., 2014). The establishment of biorepositories is expected to help improve biomedical research across Africa through the safe storage and retrieval of samples, and the opportunity for scientists, both within Africa and abroad, to have access to these samples for follow-up studies (Adoga et al., 2014, H3Africa Consortium et al., 2014). For example, it will improve accessibility to experimental samples from trusted sources thus providing a more enabling environment for developing novel approaches to address health challenges in Africa.

With the ongoing improvements in the quality of research infrastructure, more advanced experiments can now be performed in Africa, eliminating the previous burden of having to send samples abroad for analysis, thereby saving time and money. Importantly, this will also have a positive impact on student training, as many of the next generation of scientists can be trained within Africa. Moreover, these improvements will help to establish a position for African researchers beyond the level of sample collection in international collaborative projects to becoming more involved in research design, experimentation, data analysis and manuscript preparation (H3Africa Consortium et al., 2014). Altogether, by addressing the limitations that previously prevented many African scientists from effectively participating in the bioinformatics agenda, the recent developments are helping to build the foundations for potentially more fruitful bioinformatics education and research, which will contribute significantly to advancing biomedical research in Africa.

## 3 Research funding

A key challenge facing biomedical research in Africa is the lack of funding, which has had many adverse consequences on the continent's scientific development (Bishop et al., 2015, H3Africa Consortium et al., 2014, Karikari, 2015a, Karikari and Quansah, 2015, Mohamed, 2015, Quansah and Karikari, 2015). An important initiative that has recently come to support biomedical research in Africa is the Human Heredity and Health in Africa (H3Africa, www.h3africa.org/) initiative, which is poised to enrich genomics, genetics and other health-related research in order to gain better understanding of health and diseases affecting African populations. Co-funded by the National Institutes of Health (USA) and the Wellcome Trust (UK), H3Africa is one of the biggest Africa-focused biomedical research-support projects in history (Adoga et al., 2014, H3Africa Consortium et al., 2014, Karikari, 2015a). Projects funded under H3Africa and similar schemes have been highlighted in Table 1.

Bioinformatics research funding from local and national agencies remains a challenge in many parts of Africa (Adoga et al., 2014, H3Africa Consortium et al., 2014, Karikari and Quansah, 2015, Mohamed, 2015). However, a few countries have made considerable efforts to improve the situation. A typical example can be found in South Africa where genomics research benefits from government-sponsored grants from the National Research Foundation and other agencies (Pohlhaus and Cook-Deegan, 2008). With dedicated public support for scientific research, it is therefore not surprising that South Africa is a leader in bioinformatics research in Africa (Bishop et al., 2015, Karikari, 2015a). Emulating the example of South Africa, by allocating more public funds for supporting bioinformatics research would contribute immensely to the improvement of the field in other African countries (Karikari, 2015a).

## 4 Training programmes

Sustainable improvements in bioinformatics research in Africa would require that more Africa-based scientists are trained to become experts in the discipline (H3Africa Consortium et al., 2014). In this regard, various bioinformatics capacity-building programmes have been introduced in different countries. An example is the introduction of research fellowships to challenge professionals to generate more bioinformatics-motivated solutions to scientific challenges in Africa (Table 1). These advanced training schemes are expected to produce experts who would lead the anticipated boom in genetic and genomic research in Africa (Bishop et al., 2015, H3Africa Consortium et al., 2014, Karikari, 2015a).

A major advantage of bioinformatics is the availability of several free and open source resources (FOSRs) (Karikari, 2015a). The improved Internet connectivity in Africa has provided increased opportunities for scientists to access FOSRs and other databases

(Karikari, 2015a, Karikari, 2015b, Bishop et al., 2015). Additionally, the accessibility of online training programmes such as those from Coursera (https://www.coursera.org) and edX (https://www.edx.org) means that more learners can now acquire world-class education regardless of their location. Moreover, some leading universities make their bioinformatics courses available online (Table 1), allowing more learners to keep up-to-date with progress in the field. In addition, many leading scientific organisations also regularly organise bioinformatics training workshops for African peers (Table 1). These organisations include the African Society for Bioinformatics and Computational Biology (ASBCB, http://www.asbcb.org), International Society for Computational Biology (ISCB, http://www.iscb.org), the H3Africa nodes and Teaching and Research in Natural Sciences for Development in Africa (TReND in Africa, http://trendinafrica.org) (Bishop et al., 2015, Fatumo et al., 2014, Karikari, 2015a).

Several African universities have come to accept the importance of bioinformatics and this has led to the establishment of many training programmes, including short courses, workshops, and degree programmes (Bishop et al., 2015, Fatumo et al., 2014, Karikari, 2015a). The short courses provide introductory knowledge to beginners and help to improve the number of scientists with knowledge of bioinformatics (Karikari, 2015a). This enables the application of computational tools and techniques for solving biological challenges through interdisciplinary approaches (Karikari, 2015b). Furthermore, a few bioinformatics degree programmes have been established across the continent, helping to equip learners with in-depth bioinformatics knowledge and skills required to explore, analyse, interpret, and draw useful conclusions from biological data (Table 1). Overall, the ongoing improvements in training opportunities are helping to build the human resource base for bioinformatics research in Africa.

Apart from research funding, some of the international funding schemes (such as H3Africa) do provide their awardee scientists with administrative support, scientific consultation and advanced training from international leaders in bioinformatics and related areas, thus helping to build the overall expertise of these investigators (Adoga et al., 2014). Moreover, H3ABioNet, a pan-African bioinformatics network funded under the H3Africa scheme, recently established an educational committee tasked to develop curriculum guidelines for bioinformatics training in Africa (Bishop et al., 2015). This curriculum development project is expected to make important contributions towards expert training in the region. The H3ABioNet nodes in the various countries also periodically organise training programmes aimed at building the computational expertise of scientists (Bishop et al., 2015, Karikari, 2015a). Through similar initiatives, H3ABioNet aims to build the resources (both human and infrastructural) for advancing bioinformatics research in Africa (Bishop et al., 2015).

## 5 Scientific networking and collaborations

Networking presents opportunities for peer support, collaborative research and student training among bioinformatics researchers (Karikari, 2015a). Some recent developments in bioinformatics in Africa are supporting the establishment and strengthening of scientific networks. For instance, H3Africa is funding the expansion of H3ABioNet which, among other things, aims to build interactive frameworks between bioinformatics-related researchers in Africa (Bishop et al., 2015). Through this, H3ABioNet hopes to institutionalise regular channels of communication and partnership among African scientists, to enable them pool ideas and resources together to address research questions (Bishop et al., 2015, H3Africa Consortium et al., 2014). Importantly, such initiatives will support conferences, seminars, and similar platforms in African countries, during which recent advances in research would be shared. Other bioinformatics-related societies providing similar opportunities include the ASBCB, ISCB, ISCB-Regional Student Groups, African Society for Human Genetics and national networks such as those in South Africa and Nigeria (Bishop et al., 2015, Fatumo et al., 2014, Ojo and Omabe, 2011). Progress in scientific networking is helping to expand the culture of collaboration, thus helping to address research questions more effectively from interdisciplinary perspectives.

## 6 Conclusion

Recent improvements in essential resources, training opportunities, research funding and scientific networking are making important contributions to developing bioinformatics as a discipline with enormous benefits in Africa. Although many of these efforts are still in their early stages, they are ultimately expected to culminate in many positive implications for biomedical research, helping to unravel many of the currently unknown aspects of health and disease, especially diseases that appear peculiar to Africa. As avowed in the African saying that “*Thunder is not yet rain*”, it is just the beginning and a lot more needs to be done. For instance, long term sustenance of these efforts will require further commitments from all stakeholders, especially local and national research funding agencies, scientific institutions, scientists, students and the general public. We take this opportunity to appeal to African governments, parastatal and private organisations to pay more attention to and invest in bioinformatics and scientific research at large, as scientific advancements are developmental tools that can drive socioeconomic development on the continent. Conversely, the scientific community also needs to better engage the public and policymakers about the value of their research, and the need to invest further in it. Furthermore, there is the need for the establishment of more biorepositories and databases to help boost genomic research, overcome geographical barriers and share resources between African scientists. Finally, we congratulate bioinformatics-related scientific societies and networks in Africa such as the AfSHG, ASBCB and H3ABioNet for their distinguished efforts in helping to build a sustainable scientific workforce for bioinformatics education and research on the continent.

## Competing interests

The authors declare that there is no conflict of interest.

## Figures and Tables

**Table 1:** Initiatives promoting bioinformatics development in Africa⁎.

Area	Support programmes	Examples	References	
Training programmes	Short courses and workshops	The following organisations regularly organise bioinformatics courses for African scientists: ASBCB, ISCB, H3ABioNet, TReND in Africa	(Bishop et al., 2015, Karikari, 2015a, Karikari et al., 2015, Karikari and Aleksic, 2015)	
University degree programmes	Bioinformatics degree programmes have been introduced in different countries, including Egypt, Mauritius, Mali, Kenya, Nigeria, South Africa, and Tunisia	(Bishop et al., 2015, Fatumo et al., 2014, Machanick and Bishop, 2015, Ojo and Omabe, 2011)	
Fellowships	The Wellcome Trust supports training in tropical medicine through fellowships in public health and tropical medicine (for master's, doctoral, intermediate, and senior researchers). The Postgraduate Academic Mobility for African Physician–Scientists (PAMAPS) scheme is helping to build capacity for medical research.	http://www.wellcome.ac.uk/Funding/Biomedical-science/Funding-schemes/Fellowships/Public-health-and-tropical-medicine/index.htm;http://www.pamaps.net	
Curriculum development and integration	The H3ABioNet bioinformatics educational committee is developing curriculum guidelines to support bioinformatics training in Africa	(Bishop et al., 2015)	
Research infrastructure	Bioinformatics-related research institutes and centres	New bioinformatics-related research centres and institutes established in Africa include: Genomics Research Institute, Pretoria, South Africa; African Collaborative Centre for Microbiome and Genomics Research, Nigeria; West African Bioethics; H3Africa biorepository development project, South Africa; African Centre of Excellence for Genomics of Infectious Diseases, Nigeria; and the African Centre of Excellence in Bioinformatics, Mali. Several bioinformatics units and departments have also been established in the H3ABioNet nodes in several African countries.	(Bishop et al., 2015, Fatumo et al., 2014, H3Africa Consortium et al., 2014, Karikari, 2015a)	
Biorepositories	H3Africa is funding the establishment of biorepositories to support genomic research.	(Adoga et al., 2014, H3Africa Consortium et al., 2014)	
Research funding	International funding schemes	The H3Africa programme has been funding research in Africa in areas such as neglected tropical diseases, communicable and non-communicable diseases, neurological diseases and bioethics.	(Adoga et al., 2014, Bishop et al., 2015, H3Africa Consortium et al., 2014, Karikari, 2015a)	
National research funding	South Africa's National Research Foundation and Medical Research Council provide funding for genomics research projects.	(Pohlhaus and Cook-Deegan, 2008)	
Networking	Bioinformatics networks	H3Africa is funding the establishment of a bioinformatics scientific network, H3ABioNet.	(Adoga et al., 2014, Bishop et al., 2015, H3Africa Consortium et al., 2014)	
Scientific societies	The AfSHG, ASBCB, ISCB, ISCB-RSG, and local/national societies	(Bishop et al., 2015, Fatumo et al., 2014, Ojo and Omabe, 2011)	

⁎ AfSHG, African Society for Human Genetics; ASBCB, African Society for Bioinformatics and Computational Biology; ISCB, International Society for Computational Biology; ISCB — RSG, International Society for Computational Biology — Regional Student Group.
